# Metabolically healthy obese and metabolic syndrome of the lean: the importance of diet quality. Analysis of MAGNETIC cohort

**DOI:** 10.1186/s12937-020-00532-0

**Published:** 2020-02-25

**Authors:** Kamila Osadnik, Tadeusz Osadnik, Marta Lonnie, Mateusz Lejawa, Rafał Reguła, Martyna Fronczek, Marcin Gawlita, Lidia Wądołowska, Mariusz Gąsior, Natalia Pawlas

**Affiliations:** 1 Department of Pharmacology, Faculty of Medical Sciences in Zabrze, Medical University of Silesia, Jordana 38, 41-808 Zabrze, Poland; 2 2nd Department of Cardiology and Angiology, Silesian Center for Heart Diseases, Marii Skłodowskiej-Curie 9, 41-800 Zabrze, Poland; 3 Department of Human Nutrition, Faculty of Food Science, University of Warmia and Mazury in Olsztyn, Słoneczna 45f, 10-718 Olsztyn, Poland; 4 3rd Department of Cardiology, Faculty of Medical Sciences in Zabrze, Medical University of Silesia, Marii Skłodowskiej-Curie 9, 41-800 Zabrze, Poland; 5 Department of Medical and Molecular Biology, Faculty of Medical Sciences in Zabrze, Medical University of Silesia, Jordana 19, 41-808 Zabrze, Poland; 6 Department of Environmental Medicine and Epidemiology, Faculty of Medical Sciences in Zabrze, Medical University of Silesia, Jordana 19, 41-808 Zabrze, Poland

**Keywords:** Dietary patterns, Diet quality, Metabolic health, Metabolic syndrome, Principal component analysis, Young adults

## Abstract

**Background:**

Obesity is considered as an indispensable component of metabolic health assessment and metabolic syndrome diagnosis. The associations between diet quality and metabolic health in lean, young adults have not been yet established whilst data addressing this issue in overweight and obese subjects is scarce. Our analysis aimed to establish the link between diet quality (measured with data-driven dietary patterns and diet quality scores) and metabolic syndrome (MS) in young adults, regardless of their adiposity status.

**Methods:**

A total of 797 participants aged 18–35 years old were included in the study. Participants were assigned into metabolic syndrome (MS) group if at least two abnormalities within the following parameters were present: blood pressure, triglycerides, total cholesterol, HDL cholesterol, blood glucose. Participants with one or none abnormalities were considered as metabolically healthy subjects (MH), Diet quality was assessed with two approaches: 1) a posteriori by drawing dietary patterns (DPs) with principal component analysis (PCA) and 2) a priori by establishing diet quality scores and the adherence to pro-Healthy-Diet-Index (pHDI) and non-Healthy-Diet-Index (nHDI). Logistic regression with backward selection based on Akaike information criterion was carried out, to identify factors independently associated with metabolic health.

**Results:**

Within the MS group, 31% were of normal weight. Three PCA-driven DPs were identified, in total explaining 30.0% of the variance: “Western” (11.8%), “Prudent” (11.2%) and “Dairy, breakfast cereals & treats” (7.0%). In the multivariate models which included PCA-driven DPs, higher adherence to middle and upper tertiles of “Western” DP (Odds Ratios [OR] and 95% Confidence Intervals [95% CI]: 1.72, 1.07–2.79 and 1.74, 1.07–2.84, respectively), was associated with MS independently of clinical characteristics including BMI and waist-hip ratio (WHR). Similar results were obtained in the multivariate model with diet quality scores - MS was independently associated with higher scores within nHDI (2.2, 0.92–5.28).

**Conclusions:**

Individuals with MS were more likely to adhere to the western dietary pattern and have a poor diet quality in comparison to metabolically healthy peers, independently of BMI and WHR. It may imply that diet composition, as independent factor, plays a pivotal role in increasing metabolic risk. Professional dietary advice should be offered to all metabolically unhealthy patients, regardless of their body mass status.

## Introduction

Despite historical disparities in defining metabolic syndrome (MS), the commonly accepted description is the occurrence of obesity, clustered with two or more metabolic abnormalities such as hypertriglyceridemia, reduced high-density cholesterol (HDL), raised blood pressure and/or elevated fasting blood glucose levels [1]. However, the necessity of the obesity criterion in the metabolic health assessment has lately raised controversies. In the Italian cohort, Buscemi et al. [2] observed, that while 27.4% of the overweight-obese participants were metabolically healthy, 36.7% of the normal-weight participants were metabolically unhealthy. Therefore, along with the concept of metabolically healthy obese, a concept of the lean MS has emerged [3–5]. To date, many authors attempted to describe the problem, referring to this specific subpopulation as ‘normal weight obese’ [6], ‘metabolically obese nonobese’ [7], ‘thin on the outside fat on the inside – TOFI’ [8], or ‘metabolically unhealthy normal weight’ [9]. The phenomenon is being explained by the excessive and metabolically active visceral adipose tissue (VAT) [10]. The VAT is not always reflected in the BMI or waist circumference parameters, which remain within normal ranges [6–9]. Hence, the assessment of abdominal obesity based on the most commonly used measures has been identified as a serious ‘missing risk’ in patients’ diagnosis [8]. Furthermore, recent studies have shown, that patients with MS are at higher risk of developing diabetes [5], coronary heart disease [11] or stroke [12], as compared to their metabolically healthy obese counterparts. In contrary, the obese, but metabolically healthy patients are at lower risk of developing heart disease or diabetes than lean or overweight subjects with at least two mentioned abnormalities [5, 13].

High prevalence of MS in lean patients and a relatively high percentage of metabolically healthy patients in the obese population suggests that apart from overall caloric load, diet quality might be one of the independent predictors of metabolic health [14–17]. Diet is one of the major modifiable risk factor contributing to the development of chronic diseases [18]. Particularly detrimental pattern linked to MS is being described as the ‘western’ pattern; defined by a high intake of red and processed meats, fast foods, refined grains, desserts, sweets [19]. The latter relationship is well documented in multiple studies [20–22] and confirmed in the recently published meta-analyses by Rodríguez-Monforte et al. [23] and Shab-Bidar et al. [24]. To our knowledge, no previous studies used two different approaches in assessing diet quality in the context of metabolic health in a group of young adults.

Our analysis aimed to establish the link between diet quality (measured with data-driven dietary patterns and diet quality scores) and MS in young adults, regardless of their adiposity status.

## Methods

### Ethical approval

The study was conducted following the Declaration of Helsinki and good clinical practice. The study protocol has been approved by the Ethics Committee at the Institute of Occupational Medicine and Environmental Health, Sosnowiec and Medical University of Silesia. Informed written consent was obtained from all subjects enrolled in the study.

### Study sample and recruitment

**Fig. 1:**
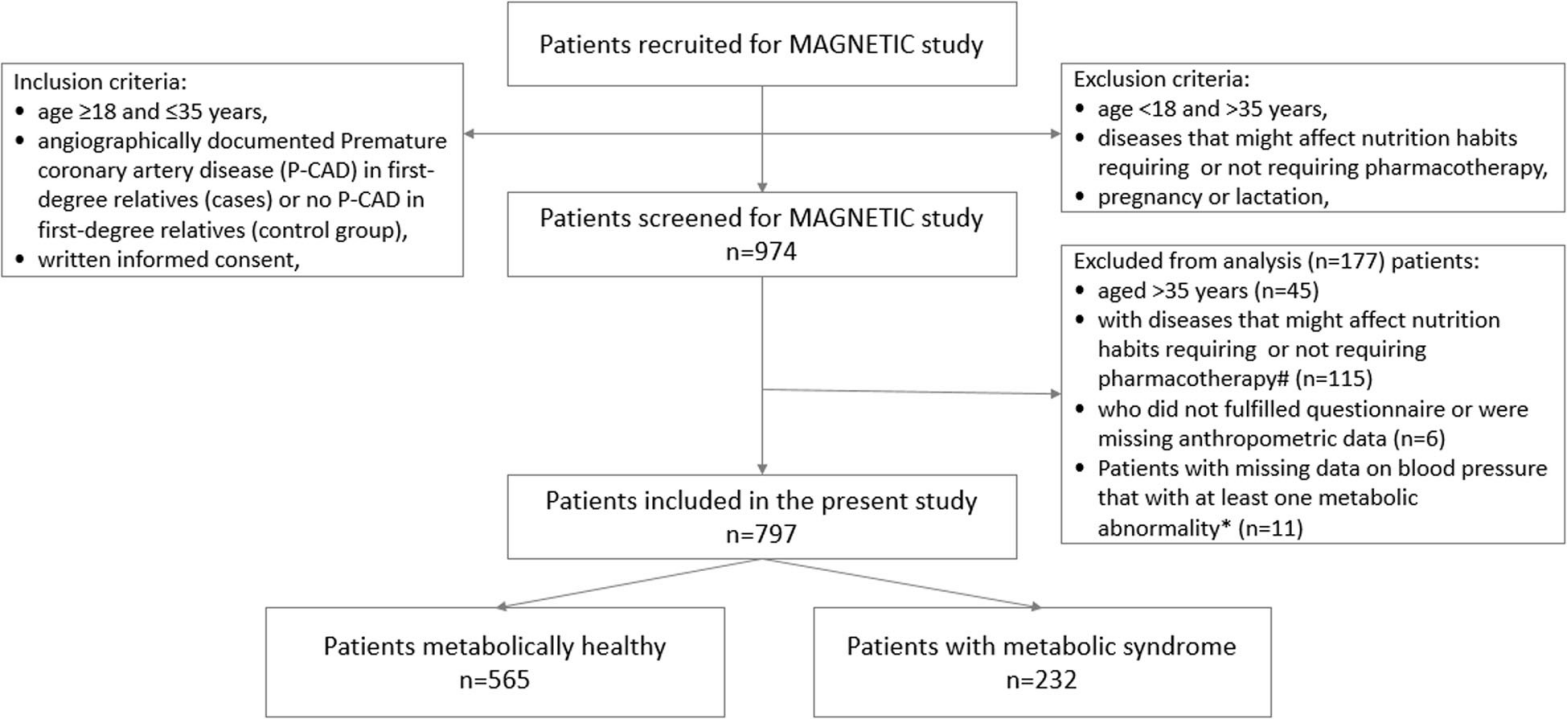
Study flow-chart. Notes: # Asthma or allergies (14), Atopic skin disease (2), Bipolar disorder or depression (3), Cholelithiasis (1), Chronic gastritis (1), Coeliac disease (1), Collitis (2), Crohn disease (1), Diabetes mellitus (1), Epilepsy (2), History of gastric ulcers (1), GERD (7), GOUT (2), Hashimoto disease and hypothyroidism (31), Hypercholesterolemia, treated with statins (1), Hyperprolactynemia (1), Hypertension (13), Idiopathic purpura (1), Irritable bowel syndrome (5), Lactation (1), Lactose intolerance (1), Marfan disease (1), Migrains (3), Nephrolithiasis, non-infectious hepatitis (1), Polycystic ovarian disease (9), Psoriasis (2), Steatosis hepatitis (1), Virial hepatitis (3). * There were 44 patients with missing data on BP, however only 11 of them had one metabolic abnormality, in which case elevated BP could change group classification (metabolically healthy vs. MS), therefore those 11 patients were excluded from analysis

Participants in this study were subjects from the MAGNETIC (Metabolic and Genetic Profiling of Young Adults with and without a Family History of Premature Coronary Heart Disease) study. The design and methodology of the study have been described previously [25, 26]. For the purpose of the current study, the sample was recruited between July 2015 and December 2017 (Fig. 1).

Since the primary aim of the MAGNETIC project was to analyse classical, genetic and metabolic risk factors of coronary artery disease (CAD) in healthy young adults with and without a family history of premature coronary artery disease (P-CAD), the inclusion criteria were: age ≥ 18 and ≤ 35 years old, angiographically documented P-CAD in first-degree relatives (cases) or no P-CAD in first-degree relatives (control group). The exclusion criteria were: age < 18 or > 35 years, failure to provide informed consent, pregnancy, lactation and acute or chronic diseases requiring pharmacotherapy. Subjects with a positive family history of P-CAD were recruited among young healthy patients of Silesian Centre for Heart Disease, and through contacting offspring of older patients hospitalized in 2010–2017 due to P-CAD. The control group was recruited from healthy subjects aged 18–35 years, who attended screening appointment at the centre.

### Metabolic health

Resting blood pressure was measured in a sitting position with the use of an automated blood pressure monitor. Fasting plasma glucose, and serum levels of total cholesterol, HDL cholesterol and triglycerides were obtained after requested 12 h fast. Next, based on the metabolic health criteria proposed by Buscemi et al. [2], the sample was divided into two distinct groups: metabolically healthy subjects – individuals without MS and subjects with MS (Table 1). Anthropometric measurements (height, weight, and waist circumference) were obtained during the first appointment at Silesian Centre for Heart Disease. Body mass index (BMI) was calculated to assess general adiposity. To classify participants as normal weight and overweight or obese within the MH and MS groups, BMI was used as categorical variable, in accordance to WHO cut-offs point: normal weight (18.5 to 24.99 kg/m2), overweight (25 to 29.99 kg/m2) and obese (≥30 kg/m2) [27]. Waist-Hip Ratio (WHR) was calculated to assess central fat distribution. WHR and WC were used as continuous variables and interpreted as follows: the higher value, the greater proportion of abdominal fat.

**Table 1:** Definition of metabolic health [2]

Parameter	Cut-offs
Blood pressure	SBP ≥130 mmHg or DBP ≥85 mmHg
	or use of antihypertensive medication^a^
Triglycerides	≥150 mg/dl
	or use of lipid-lowering medication^a^
HDL cholesterol	Men < 40 mg/dl (1.0 mmol/l)
	Women < 50 mg/dl (1.2 mmol/l)
Total cholesterol	> 200 mg/dl (5.2 mmol/l)
	or use of cholesterol-lowering medication^a^
Glucose	Glucose > 100 mg/dl (> 5.55 mmol/l) or diabetes mellitus type 2
**Metabolically healthy** (without metabolic syndrome)	0–1 of the above cut-offs
**Metabolically unhealthy** (with metabolic syndrome)	≥2 of the above cut-offs

^a^None of the subjects was on lipid lowering or hypertensive therapy

### Diet quality

Considering that human diet is a cluster of various dietary behaviours, two approaches have been adopted to investigate the associations between diet and metabolic abnormalities: a priori determined with diet quality scores and a posteriori as the adherence to data-driven dietary patterns, which represent the overall diet of studied populations [28].

Two validated food frequency questionnaires were used to assess diet quality. For data-driven dietary pattern analysis (a posteriori approach), data was collected using validated for Polish population food frequency questionnaire (FFQ-6) [29]; the self-administered version of FFQ-6 was used. The questionnaire was previously used in various populations [30–32], with the reproducibility being recently described in detail elsewhere [29]. To analyse diet quality expressed in scores (a priori approach), dietary data was collected using KomPAN questionnaire [33, 34]. Detailed description regarding reproducibility and validity of the questionnaire has been previously described [34]. Enrolled participants received questionnaires from trained researches, who provided guidance and assistance as required, on the one-to-one basis. Questionnaires were completed and returned along with signed informed consents, prior to further data collection.

### Dietary data for PCA-driven dietary patterns

FFQ-6 includes a comprehensive variety of foods (62 food items) usually consumed in Poland [29]. For the purpose of this study 46 food items were considered including ‘fruit in total’ and ‘vegetables in total’, and excluding single fruit and vegetable items. Participants could choose one of six categories, ranging from ‘never or very rarely’ to ‘few times a day’. The frequencies of consumption were converted into numerical values and expressed as times/day as follows: ‘never or very rarely’ = 0; ‘once a month or less’ = 0.025; ‘several times a month’ = 0.1; ‘several times a week’ = 0.571; ‘daily’ = 1; ‘few times a day’ = 2 [29]. Some of the food items were further combined by summing their daily frequency consumption (times/day). In total, 26 foods or food groups were included in the multicomponent analysis to identify dietary patterns (Additional file 1: Table S1).

### Dietary data for diet quality scores

Diet quality scores were calculated according to manual of KomPAN questionnaire based on the usual food frequency consumption of 24 food items over the past year [33, 34]. Participants could choose one of six categories: never, 1–3 times a month, once a week, a few times a week, once a day or few times a day. The frequencies were converted into daily frequency (times/day) with numerical values assigned as follows: 0, 0.06, 0.14, 0.5, 1 and 2, respectively. Next, two diet quality scores were used: pro-Healthy-Diet-Index (pHDI) and non-Healthy-Diet-Index (nHDI). The pHDI included 10 food items: wholemeal bread/bread rolls, coarse-ground groats, milk, fermented milk drinks, cheese curd products, white meat, fish, legumes, fruit and vegetables. The nHDI included 14 food items: white bread and bakery products, white rice and fine-ground groats, fast foods, fried foods, butter, lard, cheese, cured meat/smoked sausages/hot-dogs, red meat, sweets, tinned meats, sweetened carbonated or still drinks, energy drinks, alcoholic beverages (Additional file 1: Table S2). Daily frequencies of the consumption of the selected food items (10 items for pHDI and 14 items for nHDI) were summed up and recalculated into ranges from 0 to 100% points according to questionnaire’s manual [33, 34].

### Other factors

#### Sociodemographic

Data regarding sociodemographic variables were obtained using KomPAN questionnaire, described earlier (“Diet quality” section) [33]. Age (in years) and sex were recorded. Place of residence, level of education, financial situation collected using closed structured questions. Next, based on respondents’ declarations, dichotomous categories were created as follows: place of residence (village/city < 20,000 inhabitants vs*.* city > 20,000 inhabitants), education (higher vs primary/lower secondary or secondary), financial situation (average/below average vs. above average) [33].

#### Lifestyle

Data regarding lifestyle variables were collected with KomPAN questionnaire and included: physical activity at leisure time, smoking and smoking status [33]. Physical activity at leisure time was assigned using three categories based on intensity: low (sitting, screen time, reading, light housework, walking less than 2 h a week), moderate (walking, cycling, moderate exercise, working at home or other light physical activity performed 2–3 h/week) or high (cycling, running, working at home or other sports activities requiring physical effort over 3 h/week). Then, dichotomous categories for the purpose of multivariable analyses were created as follows: physical activity at leisure time (low or moderate vs. high), smoking (non-smoker or past smoker vs. current smoker).

#### Family history

Family history of diabetes in 1st and 2nd degree relative(s) was investigated during medical interviews during the first appointment at the centre. P-CAD in 1st degree relative(s) was confirmed angiographically.

#### Nutrition knowledge

Nutrition knowledge was assessed using KomPAN questionnaire [33]. Participants’ had to provide answers (true/false/unsure) to the set of 25 statements; 1 point was or assigned for every correct answer, and 0 points for the wrong answer or “unsure”. Next, all points were summarized to express nutrition knowledge score in points.

### Statistical analysis

Data was presented as sample percentages (%) and means with standard deviations (SDs) for variables with a normal distribution (e.g. regarding metabolic health) or medians with interquartile range (IQR) for variables without normal distribution (e.g. regarding food frequency consumption). Differences between groups were verified with Student’s t-test for means or Mann-Whitney-U test for medians or chi-square test for percentage distribution.

Principal component analysis (PCA) was used to derive PCA-driven dietary patterns. To derive dietary patterns (DPs), the frequency of consumption of 26 food groups (times/day) was standardized so that values had a mean of 0 and a standard deviation of 1. PCA with promax rotation was used. Components to retain were based on their interpretability and eigenvalues (> 1) and a break-point identified in the Scree test. The contribution of each questionnaire item to each DP is reflected by the item’s factor loading. Factor loadings >|0.30| were considered to be of significant contribution to identified DPs. Dietary patterns were labelled according to variables with the highest loadings for each dietary pattern. For each subject, a DP score that reflects adherence to the DP was calculated (as a sum of the product of the food frequency consumption and factor loading for 26 food groups). Based on tertile distribution, for each DP subjects were divided into three groups: bottom, middle and upper tertile and interpreted as lowest, moderate and highest adherence to the DP, respectively.

Unconditional logistic regression analysis was used to assess the association between metabolic health status and adherence to identified DP or diet quality scores [35]. Univariate and multivariate logistic regression with backward selection models were carried out, to identify factors independently associated with metabolic health status. Factors that were kept in the multivariate model were based on the Akaike information criterion. Variables that were initially included in model were as continuous variables: age, BMI, WHR, and as categorical variables: sex, place of residence, financial situation, education, smoking status, physical activity at leisure time, family history of diabetes mellitus in 1st and 2nd degree relative(s), family history of P-CAD in 1st degree relative(s) as well as PCA-driven DPs (categorical variables) and diet quality scores (continuous variables). Two separate multivariate models were built for tertile intervals of each DP calculated for dietary pattern scores, and for models incorporating pHDI and nHDI (both in % points).

There was a total of 0.8% of missing values. Before performing multivariable logistic regression analysis missing values were imputed using the missForest data imputation algorithm. For all tests, the *P*-value of < 0.05 was considered to be statistically significant.

## Results

### Sample characteristics

In comparison to the MH group, the MS group had a higher mean BMI (23.0 ± 3.5 vs. 27.8 ± 4.6 kg/m2, respectively), WHR (0.82 ± 0.09 vs. 0.90 ± 0.08, respectively) and WC (85.1 ± 9.0 vs. 92.9 ± 11.6 and 72.2 ± 9.0 vs. 89.3 ± 14.8, for men and women respectively) (Table 2). Within the MH group, approx. 73% of the subjects were normal weight and approx. 27% were overweight or obese. Within the MS group, approx. 31% of subjects were normal weight and 69% were overweight or obese. Patients from the MS group were older (29.4 ± 4.1 vs. 27.2 ± 4.5 years), more often males. A higher percentage of patients with a family history of P-CAD was found in the MS then MH group (57.8% vs. 46.0%). Also, the nutrition knowledge was lower in the MS group, in comparison to the MH group (11.5 ± 4.0 vs. 12.5 ± 3.9 points, respectively). There were no differences between MH and MS subjects with regards to the place of residence, education level, financial situation, nutrition knowledge score, physical activity level, smoking status, family history of diabetes mellitus. Blood pressure, glucose and lipid parameters were significantly different between the groups (Table 2).

**Table 2:** Characteristics of study participants by metabolic health status (number (%) or mean ± standard deviation or median ± interquartile range)

Variables	Total sample	MH	MS	*P* value
Sample size	**797**	**565**	**232**	–
Sample percentage	100	70.9	29.1	–
Sociodemographic				
Age (years)	27.9 ± 4.5	27.2 ± 4.5	29.4 ± 4.1	< 0.0001
Male sex (%)	460 (57.7)	268 (47.4)	192 (82.8)	< 0.0001
Residence (%)				
village	121 (15.2)	80 (14.2)	41 (17.7)	0.179
small town (< 20,000 inhabitants)	89 (11.2)	68 (12.0)	21 (9.1)	
town (20,000 to 100,000 inhabitants)	161 (20.2)	107 (18.9)	54 (23.3)	
city (> 100,000 inhabitants)	426 (53.5)	310 (54.9)	116 (50.0)	
Education (%)				
primary/lower secondary	53 (6.6)	29 (5.1)	24 (10.3)	0.021
upper secondary	302 (37.9)	213 (37.7)	89 (38.4)	
higher	442 (55.5)	323 (57.2)	119 (51.3)	
Financial situation				
average/below average	598 (75.1)	431 (76.4)	167 (72.0)	0.05
above average	198 (24.9)	133 (23.6)	65 (28.0)	
Nutrition knowledge score (points)	12.2 ± 3.9	12.5 ± 3.9	11.5 ± 4.0	0.0009
Lifestyle				
Physical activity at leisure				
low	206 (25.8)	134 (23.7)	72 (31.0)	0.087
moderate	366 (45.9)	264 (46.7)	102 (44.0)	
high	225 (28.2)	167 (29.6)	58 (25.0)	
Current smoking (vs. non-smoking) (%)	184 (23.3)	120 (21.4)	64 (27.8)	0.053
Family health history				
Diabetes mellitus in 1st degree relatives (%)	113 (14.2)	73 (12.9)	40 (17.2)	0.112
Premature CAD in 1st degree relatives (%)	394 (49.4)	260 (46.0)	134 (57.8)	0.003
Dietary				
pHDI (% points)	21.0 ± 10.1	21.6 ± 9.9	19.7 ± 10.6	0.005
nHDI (% points)	17.5 ± 8.1	16.7 ± 7.8	19.4 ± 8.3	< 0.0001
Lowest adherence to DP (%)				
Prudent	266 (33.4)	179 (31.7)	87 (37.5)	0.113
Western	266 (33.4)	213 (37.7)	53 (22.8)	< 0.0001
Dairy, breakfast cereals & treats	266 (33.4)	176 (31.2)	90 (38.8)	0.809
Moderate adherence to DP (%)				
Prudent	266 (33.4)	196 (34.7)	70 (30.2)	0.219
Western	266 (33.4)	183 (32.4)	83 (35.8)	0.357
Dairy, breakfast cereals & treats	266 (33.4)	196 (34.7)	70 (30.2)	0.219
Highest adherence to DP (%)				
Prudent	265 (33.2)	190 (33.6)	75 (32.3)	0.7233
Western	265 (33.2)	169 (29.9)	96 (41.4)	0.002
Dairy, breakfast cereals & treats	265 (33.2)	193 (34.2)	72 (31.1)	0.395
Adiposity				
BMI (kg/m^2^)	24.4 ± 4.4	23.0 ± 3.5	27.8 ± 4.6	< 0.0001
BMI by categories (kg/m^2^)				
normal weight	21.6 ± 2.2	21.3 ± 2.2	22.9 ± 1.7	< 0.0001
overweight	27.0 ± 1.3	26.9 ± 1.3	27.3 ± 1.4	0.01
obesity	33.4 ± 2.6	32.4 ± 2.5	33.7 ± 2.6	0.06
BMI categories (%)				
normal weight	481 (60.4)	410 (72.6)	71 (30.6)	< 0.0001
Overweight	231 (29.0)	136 (24.1)	95 (40.9)	
Obesity	84 (10.5)	18 (3.2)	66 (28.4)	
WHR	0.84 ± 0.09	0.82 ± 0.09	0.90 ± 0.08	< 0.0001
WHR men	0.89 ± 0.08	0.87 ± 0.08	0.91 ± 0.07	< 0.0001
WHR women	0.78 ± 0.07	0.78 ± 0.07	0.83 ± 0.07	< 0.0001
Waist circumference men	88.4 ± 9.0	85.1 ± 9.0	92.9 ± 11.6	< 0.0001
Waist circumference women	74.2 ± 11.3	72.2 ± 9.0	89.3 ± 14.8	< 0.0001
Metabolic health				
SBP (mmHg)	126.7 ± 14.3	122.8 ± 12.8	135.6 ± 13.7	< 0.0001
DBP (mmHg)	78.6 ± 10.8	76.4 ± 9.8	83.8 ± 11.2	< 0.0001
Total cholesterol (mmol/l)	4.94 ± 1.04	4.65 ± 0.9	5.66 ± 1.13	< 0.0001
LDL-cholesterol (mmol/l)	2.96 ± 0.9	2.67 ± 0.8	3.68 ± 0.9	< 0.0001
HDL-cholesterol (mmol/l)	1.59 ± 0.45	1.70 ± 0.41	1.32 ± 0.40	< 0.0001
Triglycerides (mmol/l)	1.20 ± 1.14	0.87 ± 0.37	1.99 ± 1.80	< 0.0001
Glucose (mmol/l)	5.00 ± 0.46	4.89 ± 0.38	5.27 ± 0.53	< 0.0001
Metabolic abnormalities (%)				
Elevated SBP	305 (39.9)	142 (26.5)	163 (71.2)	< 0.0001
Elevated DBP	213 (27.9)	99 (18.5)	114 (49.8)	< 0.0001
Elevated total cholesterol	279 (35.0)	121 (21.4)	158 (68.1)	< 0.0001
Elevated LDL-cholesterol	237 (29.7)	90 (15.9)	147 (63.4)	< 0.0001
Lowered HDL-cholesterol	62 (7.8)	8 (1.4%)	54 (23.3)	< 0.0001
Elevated triglycerides	125 (15.7)	16 (2.8)	109 (47.0)	< 0.0001
Elevated glucose	99 (12.4)	21 (3.7)	78 (33.6)	< 0.0001

### Diet quality

#### PCA-driven dietary patterns

The principal component analysis identified three dietary patterns in total explaining 30.0% of the variance (Table 3). “Western” dietary pattern was characterised by higher consumption frequency of processed meat (0.70), potatoes (0.62), refined grain products (0.56), animal fats (0.54), red meats (0.53), other edible fats (0.45), sweetened beverages and energy drinks (0.40), sugar (0.39), alcohol (0.36), cheeses (0.35) and sweets and snacks (0.35), white meat (0.30). “Prudent” dietary pattern was characterised by higher frequency consumption of wholegrain products (0.64), vegetables (0.62) fish (0.53), eggs and egg dishes (0.52), nuts and seeds (0.52), fruits (0.51), milk, fermented milk drinks and curd cheese (0.44), vegetable oils (0.41), white meat (0.39) and legumes (0.38). The third dietary pattern, “Dairy breakfast cereals & treats” was characterized by frequent consumption of sweetened milk products (0.70), milk, fermented milk drinks and curd cheese (0.54), breakfast cereals (0.49), sweets and snacks (0,47) and fruit (0.43) (Table 3).

**Table 3:** PCA-driven dietary patterns (DPs) identified in the total sample by principal component analysis: data from FFQ-6 questionnaire

Food items	Factor loadings		
	Western DP	Prudent DP	Dairy, breakfast cereals & treats DP
Processed meats	0.70		
Potatoes	0.62		
Refined grain products	0.56		
Animal fats	0.54		
Red meats	0.53		
Other edible fats	0.45		
Sweetened beverages and energy drinks	0.40		
Sugar	0.39		
Cheeses	0.35		
Vegetables		0.62	
Whole grain products		0.64	
Fish		0.53	
Eggs and egg dishes		0.52	
Nuts and seeds		0.52	
Fruit		0.51	0.43
Vegetable oils		0.41	
White meat	0.30	0.39	
Legumes		0.38	
Sweetened milk products			0.70
Milk, fermented milk drinks and curd cheese		0.44	0.54
Breakfast cereals			0.49
Sweets and snacks	0.35		0.47
Alcohol	0.36		
Variance explained (%)	11.8	11.2	7.0

Factor loadings of >|0.30| are shown in the table, therefore three dietary items (out of 26) are not presented for simplicity. Sorted by loadings from 1st to 3rd factor. Total variance in dietary variables explained by three patterns is 30.0%

#### Diet quality scores

Medians and interquartile ranges of frequency consumptions of food items within pHDI and nHDI are listed in Table 4. In total, the median of pHDI was 6.84 times/day (within the range 0–20) and median of nHDI was 4.70 times/day (within the range 0–28).

**Table 4:** Diet quality scores (in times/day) identified in the total sample with pro-Healthy-Diet-Index (pHDI) and non-Healthy-Diet-Index (nHDI) by metabolic health status: data from KomPAN questionnaire (median and interquartile range)

Diet quality scores and food items	Total sample	MH	MS	P value
pHDI	3.88 (2.7–5.32)	3.98 [2.85–5.49]	3.47 (2.48–4.82)	0.005
Components of pHDI				
(1) Fruit	0.50 (0.50–1.00)	0.50 (0.50–1.00)	0.50 (0.50–1.00)	0.059
(2) Vegetables	0.50 (0.50–1.00)	0.50 (0.50–1.00)	0.50 (0.50–1.00)	0.083
(3) Milk	0.50 (0.14–1.00)	0.50 (0.14–1.00)	0.50 (0.06–1.00)	0.031
(4) White meat	0.50 (0.14–0.50)	0.50 (0.14–0.50)	0.50 (0.14–0.50)	0.522
(5) Coarse-ground groats	0.14 (0.06–0.50)	0.14 (0.06–0.50)	0.06 (0.06–0.14)	0.003
(6) Fermented milk drinks	0.14 (0.06–0.50)	0.14 (0.06–0.50)	0.14 (0.06–0.50)	0.466
(7) Cheese curd products	0.14 (0.06–0.50)	0.14 (0.06–0.50)	0.14 (0.06–0.50)	0.598
(8) Whole meal bread	0.14 (0.06–0.50)	0.14 (0.06–0.50)	0.14 (0.06–0.50)	0.883
(9) Fish	0.06 (0.06–0.14)	0.06 (0.06–1.00)	0.06 (0.06–0.14)	0.989
(10) Legumes-based foods	0.06 (0.06–0.06)	0.06 (0.06–0.06)	0.06 (0.06–0.06)	0.118
nHDI	4.70 (3.26–6.26)	4.48 (3.08–6.08)	5.38 (3.65–6.63)	< 0.0001
Components of nHDI				
(1) White bread	0.50 (0.50–1.00)	0.50 (0.50–1.00)	0.50 (0.50–2.00)	0.171
(2) Cheese	0.50 (0.14–0.50)	0.50 (0.14–0.50)	0.50 (0.14–0.50)	0.035
(3) Cured meat	0.50 (0.14–0.75)	0.50 (0.14–0.50)	0.50 (0.50–1.00)	0.002
(4) Fried foods	0.50 (0.14–0.50)	0.50 (0.14–0.50)	0.50 (0.14–0.50)	0.025
(5) Sweets	0.50 (0.14–0.50)	0.50 (0.14–0.50)	0.50 (0.14–0.50)	0.359
(6) Butter	0.50 (0.06–1.00)	0.50 (0.06–1.00)	0.50 (0.14–1.00)	0.032
(7) Red meat	0.14 (0.06–0.50)	0.14 (0.06–0.50)	0.14 (0.06–0.50)	0.039
(8) White rice and fine-ground groats	0.14 (0.06–0.50)	0.14 (0.06–0.50)	0.14 (0.06–0.50)	0.897
(9) Sweetened carbonated or still drinks drinks	0.06 (0.06–0.50)	0.06 (0.06–0.50)	0.14 (0.06–0.50)	< 0.0001
(10) Alcoholic beverages	0.06 (0.06–0.14)	0.06 (0.06–0.14)	0.14 (0.06–0.50)	< 0.0001
(11) Fast foods	0.06 (0.06–0.14)	0.06 (0.06–0.14)	0.06 (0.06–0.14)	0.010
(12) Energy drinks	0.00 (0.00–0.06)	0.00 (0.00–0.06)	0.00 (0.00–0.06)	0.0002
(13) Lard	0.00 (0.00–0.06)	0.00 (0.00–0.06)	0.00 (0.00–0.06)	0.011
(14) Tinned (jar) meats	0.00 (0.00–0.06)	0.00 (0.00–0.06)	0.00 (0.00–0.06)	0.002

### Associations between diet and metabolic health

**Fig. 2:**
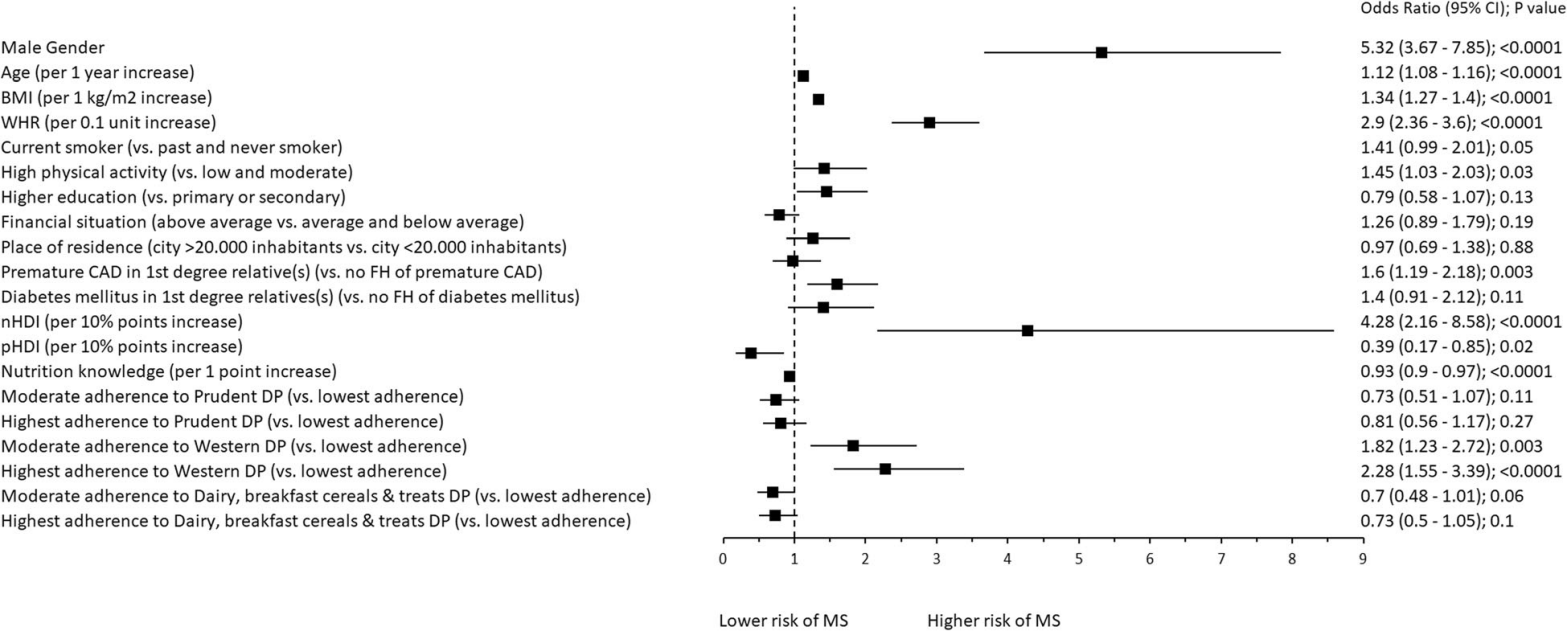
Factors associated with MS. Univariate analysis. Notes: BMI – body mass index. WHR – Waist/Hip Ratio. CAD – coronary artery disease. FH – family history. nHDI – non-Healthy-Diet-Index. pHDI – pro-Healthy-Diet-Index. DP – dietary pattern. MS – metabolic syndrome. 95% CI – 95% Confidence Interval

In the univariate analysis, higher adherence to nHDI (Odds Ratio: 4.28 per 10% points increase, 95% Confidence Interval: 2.16–8.58) and higher adherence to “Western” DP (highest vs. lowest adherence: 2.28, 1.55–3.39; moderate vs. lowest adherence: 1.82, 1.23–2.72) was associated with MS. Other significant factors associated with MS in this model included: male gender (5.32, 3.67–7.85), WHR (2.9 per 0.1 unit increase, 2.36–3.6), P-CAD in 1st degree relative(s) (vs. no P-CAD: 1.6, 1.19–2.18), high physical activity (vs. low and moderate: 1.45, 1.03–2.03), current smoking (vs. past and never smoking: 1.41, 0.99–2.01), BMI (1.34 per 1 kg/m2 increase, 1.27–1.4), age (1.12 per 1 year increase, 1.08–1.16) and nutrition knowledge score (0.93 per 1 point increase, 0.9–0.97). Whereas there is a reverse dependence of MS to adherence to pHDI (0.39 per 10% points increase, 0.17–0.85) (Fig. 2).

**Fig. 3:**
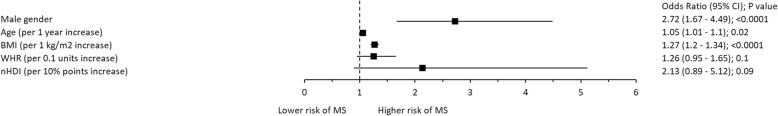
Independent factors associated with MS. Multivariate analysis with the inclusion of PCA-derived dietary patterns. Variable selection was based on Akaike information criterion. Notes: BMI – body mass index. WHR – Waist/Hip Ratio. DP – dietary pattern. MS – metabolic syndrome. 95% CI – 95% Confidence Interval

In the multivariate model which included PCA-driven DPs, individuals who showed higher adherence to the “Western” DP (highest vs. lowest adherence: 1.64, 1.02–2.64; moderate vs. lowest adherence: 1.64, 1.02–2.64, respectively) were more likely to have MS, independently of other significant factors: male gender (2.75, 1.69–4.54), BMI (1.27 per 1 kg/m2 increase, 1.20–1.34), WHR (1.22 per 0.1 unit increase, 0.92–1.61) and age (1.06 per 1 year increase, 1.01–1.11) (Fig. 3).

**Fig. 4:**
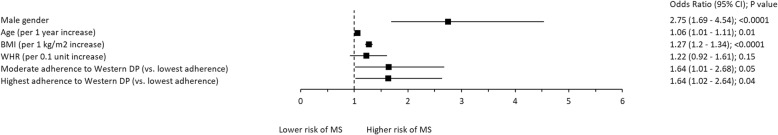
Independent factors associated with MS. Multivariate analysis with the inclusion of pro-Healthy-Diet-Index (pHDI) and non-Healthy-Diet-Index (nHDI). Variable selection was based on Akaike information criterion. Notes: BMI – body mass index. WHR – Waist/Hip Ratio. MS – metabolic syndrome. 95% CI – 95% Confidence Interval

In the multivariate model which included diet quality scores, MS was associated with higher adherence to nHDI (2.13 per 10% points increase, 0.89–5.12), independently of other significant factors: male gender (2.72, 1.67–4.49), BMI (1.27 per 1 kg/m2 increase, 1.20–1.34), age (1.05 per 1 year increase, 1.01–1.10) (Fig. 4).

## Discussion

In this study, adherence to the western dietary pattern and low diet quality were independent predictors of poor metabolic health.

Excessive body weight is considered a key modifiable risk factor associated with metabolic health and is recognized as a vital component of metabolic syndrome diagnosis in clinical practice [1]. As anticipated, depending on the model, BMI alone or both adiposity measures (BMI and WHR) were independent factors of metabolic syndrome. However, the results of our study also revealed that within the group of young people with MS, approx. 31% had BMI within a healthy range. This concurs well with previous findings. Wildman et al. [36] found, that in a Canadian cohort, nearly 25% of normal weight adults > 20 years old were metabolically abnormal. Perceiving optimal BMI as a sole metabolic health status indicator is therefore misleading and should be complemented with additional measures by health professionals, as metabolic syndrome might occur in 20–30% of young lean adults.

Obesity is the most visible feature but can be misinterpreted as a key criterion for metabolic syndrome. It is well established that the assessment of fat distribution is more crucial than total adiposity in metabolic health screening [37]. One of the measures to define the location of excessive adipose tissue is WHR [38]. This measure has shown to be an independent predictor of metabolic health in multivariate models in our study (Figs. 3 and 4). We think, that WHR is a good diagnostic indicator of central obesity but may not be a prognostic indicator of the MS in lean patients. Based on these findings, it can be concluded that in adults with metabolic abnormalities, not only body weight management, but more specific lifestyle modifications are required. Precisely, an emphasis on diet quality might be an equally important approach in metabolic health management, which should not be neglected by clinicians. While weight loss recommendation applies to overweight patients, promoting healthy dietary patterns addresses the needs of all individuals at risks, including those with normal weight [39].

We showed that an unhealthy diet is one of the critical independent predictors of metabolic abnormalities, which was confirmed with two approaches: dietary patterns analysis and as pre-defined diet quality measure, expressed as diet quality scores. Adults with metabolic abnormalities (including 31% those with normal BMI) were more likely to adhere to the “Western” dietary pattern and have a poor diet quality than metabolically healthy adults. The adherence to this pattern was be observed not only among obese, but also lean individuals. This observation supports previous reports, that the diet composition regardless of energy load, can play a significant role in increasing metabolic risk [14–17, 40]. A diet high in processed food, red meat, sugary snacks and low in fruit and vegetable, fish, and dairy was previously shown to be associated with an increased risk of metabolic syndrome [40]. Bahadoran et al. [14] found that fast-food intake was associated with increased risk of metabolic syndrome, which remained significant in the model adjusted for total energy intake and energy density, as well as several other potential confounders, such as sex, age, BMI, education, smoking, physical activity, total fibre, dietary intake of whole grains, fruits, vegetables, dairy, total meat, phytochemical index, and dietary total antioxidant capacity [14]. Interestingly, the association was the strongest in adults < 30 years old. It has been suggested, that the excessive consumption of saturated- and trans-fatty acids, sugar, salt and insufficient intake of fibre, micro/macronutrient and antioxidants can trigger pathogenic mechanisms, without affecting adiposity status [14, 17]. This effect might be somewhat masked in lean individuals, who maintain healthy daily energy balance, and as a result, do not gain weight. The importance of the ‘type of calories’ has been previously stressed by Ludwig [15], who suggested that iso-caloric diets may trigger different responses (e.g. hormonal, gene expression) depending on the diet composition. This field of research however still require more investigation [41–43].

It is well established that positive energy balance is the main cause of obesity, however recent hypothesis announced by Stanhope et al. [44] raise the aspect that various dietary components or patterns may promote obesity and cardiometabolic diseases due to other mechanisms than increased energy intake. Some evidence suggests that high-sugar foods (closely related to the Western diet) stimulate the reward system located in the central nervous system leading to secondary overeating [45, 46]. It has also been shown that sweetened beverages, especially fructose or sucrose-sweetened, increase the risk of developing cardiometabolic diseases and type 2 diabetes [44, 47–50]. In our study sweetened drinks were components of both “Western” dietary pattern and nHDI and its consumption was significantly more frequent in MS than MH subjects (data not shown). Although the mechanisms are still not fully understood, one of the potential explanation might be that non-nutritive sweeteners such as aspartame, sucralose, saccharin, acesulfame K and steviol glycosides indirectly affect energy balance [51–53]. The mechanism that may explain these association is the disturbance of sweet taste receptor activation and the expected relationship between sweetness and calories on gut microbiota [54–56]. The results of studies indicate that energy intake and changes alternate along with the gut microbiome composition [57–59].

The non-modifiable factors independently associated with the occurrence of metabolic abnormalities were male gender and age. Men were at higher risk, which can be a result of sex-related metabolic and hormonal differences, as well as psychological and lifestyle differences [60–62]. This result is in line with previous findings from the Baltic region. Mattson et al. [63] found that in Finish adults aged 24–39 the prevalence increased with age in both sexes, but more dramatically in men. In general, women tend to make more conscious dietary choices, e.g. more frequently eat fruit and vegetables, and less likely to opt for foods high in fat [64]. The potential explanation includes genetic susceptibility or lifestyle factors; often, unhealthy lifestyle habits acquired at family home, track to adulthood [26]. An interesting finding was, that in the univariate model, high level of leisure time physical activity was associated with higher risk of MS (Fig. 2). The potential explanation is, that the diagnosis was a trigger of a spontaneous positive lifestyle change, such as increasing the level of physical activity [65]. However, this association was of marginal significance (*p* = 0.03) and it was not retained in the multivariate model. Similarly, it was not significant when the percentage distribution between the two groups was compared (Table 2).

The main strength of this study is the use of two approaches in examining diet quality - PCA-driven approach and diet quality scores [28]. Perhaps, PCA-driven approach reflected more precisely the complex matrix of diet components, with a more detrimental effect on health, than the investigator-driven approach. To our knowledge, this is the first study, which investigated the associations between diet composition and metabolic health in young adults, regardless of their adiposity status. Because of case-control study design and its intrinsic limitations, a causal relationship could not be determined so prospective studies are needed to confirm the relationship and examine underlying mechanisms associated with poor dietary choices. Paradoxically, limitation of this study is also lack of widely accepted definition of MetS in normal weight patients, therefore definition used in this study may differ from future studies. Nonetheless results of our paper add another brick to discussion of metabolic abnormalities in lean patients, while definition of lean MetS is still being coined. Lastly, it can be argued that a more precise assessment of abdominal obesity could have been used, such as measurement of visceral fat tissue (VAT), e.g. using magnetic resonance imaging (MRI) or computed tomography (CT) scan dual energy x-ray absorptiometry (DEXA). Some studies suggested, that VAT has a stronger association with an unfavourable metabolic risk profile, which may not be detected with simple measurements [66]. In our study we have used simple, inexpensive anthropometric measurement, most commonly used in the clinical setting, such as BMI, WC and WHR. Interestingly, in the multivariable analysis there was association between WHR and metabolic health, whilst WC was left out of the multivariable model due to lack of statistical significance. Most probably, the reason for this was purely mathematical – although both WHR and WC alone are key indices of fat distribution, the WHR to some extent naturally corrects for sex as it includes hip circumference that is also a sex-dependent factor [67, 68]. Therefore, WHR in the context of multivariable analysis adds more information to the model than WC alone.

## Conclusions

Individuals with metabolic syndrome were more likely to adhere to the western dietary pattern and have a poor diet quality in comparison to metabolically healthy peers, independently of BMI and WHR. It implies that diet composition, independently, plays a pivotal role in increasing metabolic risk. Currently, in clinical practice mainly overweight or obese individuals are offered professional dietary advice, presuming that lean individuals do not require dietary modification. This may be detrimental for the latter group, in which the continuation of an unhealthy diet may escalate the abnormalities in the long-term perspective. Therefore, dietary advice should be offered to all metabolically unhealthy patients, regardless of their body mass status, with more focus on dietary quality than reducing the energy load. Remarkably, we found that the protective effect of a healthy diet was not as strong in comparison to the detrimental effect of an unhealthy diet. Perhaps, eliminating unhealthy dietary habits, rather than enforcing the healthy guidelines could be a more effective strategy in reducing the health risks.

## Supplementary information

**Additional file 1:Table S1.** Food frequency questionnaire food and food groups included in PCA-driven dietary patterns analysis (FFQ-6). **Table S2.** Food frequency questionnaire food and food groups included in diet quality scores analysis (KomPAN questionnaire). **Table S3.** Missing data.

**Additional file 2.** Dataset (raw data): Study dataset.

**Additional file 3.** Dataset (imputed data): Dataset with imputed values using random forest algorithm (used for multivariable analysis).

## Data Availability

All data generated or analysed during this study are included in this published article [see Additional file 2. Dataset (raw data), Additional file 3. Dataset (imputed data)].

## Abbreviations

BMI: Body mass index
CAD: Coronary artery disease
DBP: Diastolic blood pressure
DPs: Dietary patterns
FFQ-6: Food frequency questionnaire with 6 answers
HDL: High-density cholesterol
IQR: Interquartile range
KomPAN: Dietary Habits and Nutrition Beliefs Questionnaire
MAGNETIC: Metabolic and Genetic Profiling of Young Adults with and without a Family History of Premature Coronary Heart Disease Study
MH: Metabolically healthy subjects
missForest: Non-parametric missing value imputation for mixed-type data
MS: Metabolic syndrome
nHDI: Non-Healthy-Diet-Index
PCA: Principal component analysis
P-CAD: Premature coronary artery disease
pHDI: Pro-Healthy-Diet-Index
SDs: Standard deviations
VAT: Visceral adipose tissue
WHO: World Health Organization
WHR: Waist-hip ratio
