# Supplementary material for: Metabolically healthy obese and metabolic syndrome of the lean: the importance of diet quality. Analysis of MAGNETIC cohort
Source: Nutr J. 2020 Feb 25;19:19. doi: 10.1186/s12937-020-00532-0 (PMC7041188; doi:10.1186/s12937-020-00532-0)
Supplement: Supplementary file 1 — Additional file 1:Table S1. Food frequency questionnaire food and food groups included in PCA-driven dietary patterns analysis (FFQ-6). Table S2. Food frequency questionnaire food and food groups included in diet quality scores analysis (KomPAN questionnaire). Table S3. Missing data. [file 12937_2020_532_MOESM1_ESM.doc]

**Additional file 1: Table S1.** Food groups included in PCA-driven dietary patterns analysis: data from FFQ-6 questionnaire.

|  | **Food group** | **Questionnaire item(s)** | **Remarks** |
| --- | --- | --- | --- |
| 1. | Sugar | Sugar | Sugar added to beverages, such as tea, coffee, etc. |
| 2. | Honey | Honey, | Honey added to dishes and added to beverages |
| 3. | Sweets and snacks | Bakers’ confectionery | Biscuits, cream cakes, sponge cakes, cheesecakes, doughnuts, poppy-seed cakes, croissants etc. |
|  |  | Ice-creams and custard | Ice-creams and custard |
|  |  | Chocolates | Chocolate, chocolate sweets and chocolate bars |
|  |  | Sugar confectionery | Boiled sweets, hard caramels, jellied sweets, fudge, etc. |
|  |  | Savoury snacks | Crisps, crackers, pretzels |
| 4. | Milk, fermented milk drinks and curd cheese | Milk and milk beverages – natural | Milk and natural milk beverages (yoghurt, kefir, buttermilk), porridge, etc. |
|  |  | Cheese curds | Cheese curd, natural cottage cheese, soft cheese, mozzarella, cottage cheese with herbs, etc. |
| 5. | Sweetened milk products | Milk beverages – sweetened | Fruit yoghurts, yoghurts with chocolate flakes, flavoured buttermilk, hot chocolate etc |
|  |  | Flavoured cheese curds | Flavoured curds (with fruit, chocolate, vanilla), etc. |
| 6. | Chesses | Cheese | Hard cheese, blue cheese, processed cheese, cheese spreads, etc. |
| 7. | Eggs and egg dishes | Eggs and egg dishes | Scrambled eggs, omelette, egg salad, cooked eggs |
| 8. | Breakfast cereals | Breakfast cereals | Muesli, cornflakes, other cereals - sweetened or unsweetened, etc. |
| 9. | Whole grain products | Wholemeal cereals | Wholemeal wheat or rye bread, seeded loafs, pumpernickel, wholemeal cracker bread, etc. |
|  |  | Coarse groats | Buckwheat groats, barley, brown rice, wholemeal pasta, etc. |
| 10. | Refined grain products | Refined cereals | White bread, rye, wheat-rye bread, toast bread, white bread rolls, brioche, bagels, etc. |
|  |  | Fine groats | Semolina, milled barley, pasta, white rice, rice flakes, etc. |
| 11. | Animal fats | Butter | Butter |
|  |  | Cream | Single, double, sour, used as an ingredient or added to beverages |
|  |  | Other animal fats | Lard, pork fat, etc. |
| 12. | Red meats | Red meat | Pork, beef, veal, etc. |
| 13. | Venison | Venison | Wild boar, venison, quail, mallard, hare, etc. |
| 14. | Processed meats | Sausages, bacon, reconstituted meat | Sausages, meat loaf, hot-dogs, smoked sausages, bacon, etc. |
|  |  | High quality cured meats | Ham, poultry and pork-beef good quality cold meats, etc. |
|  |  | Offal products | Liver, blood sausage, sweetbread, liver pate, etc. |
| 15. | Vegetables | All kind of vegetables (potatoes not included) | All kind of vegetables (potatoes not included) |
| 16. | Potatoes | Potatoes | Boiled, baked, French fries, potato rosti, gnocchi, etc. |
| 17. | Vegetable oils | Vegetable based oil |  |
| 18. | Other edible fats | Margarine | Margarine for baking, frying, spreading |
|  |  | Mayonnaise | Mayonnaise and salad dressings |
| 19. | White meat | Poultry and rabbit |  |
| 20. | Fish | Lean fish | Pollock, cod, perch, hake, carp to 1 kg, tuna, panga, trout etc. |
|  |  | Oily fish | Salmon, sardines, herring, mackerel, eel, large carp etc. |
| 21. | Fruits | All kind of fruits | All kind of fruits |
| 22. | Nuts and seeds | Nuts and nut spreads | Peanuts, hazelnuts, walnuts, cashews, coconuts, chestnuts etc. |
|  |  | Seeds and bran | Pumpkin seeds, sesame seeds, sunflower seeds, wheat germs, wheat bran, etc. |
| 23. | Legumes | Fresh and tinned legumes | Corn, green peas, green beans, etc. |
|  |  | Dry and processed pulses | Beans (fava, butter kidney, broad, French, green), soya, peas, chickpea and processed pulses (baked beans, hummus, other bread spreads) |
| 24. | Juices | Fruit juices and nectars | Mixed fruit juice, orange, grapefruit, apple, pear, grape, blackcurrant, cherry juice |
|  |  | Vegetable and vegetable-fruit juices | Mixed vegetable juice, tomato, carrot and carrot-fruit juice |
| 25. | Sweetened beverages and energy drinks | Sweetened beverages |  |
|  |  | Energy drinks |  |
| 26. | Alcohol | Beer | Beer |
|  |  | Wine and cocktails | Wine and cocktails |
|  |  | Spirits | Vodka and other spirits |

| **Additional file 1: Table S2.** Food groups included in diet quality scores analysis: data from KomPAN questionnaire. | |
| --- | --- |
| **Components of pro-Healthy-Diet-Index (pHDI)** | |
| (1) | Wholemeal (brown) bread/bread rolls |
| (2) | Buckwheat, oats, wholegrain pasta or other coarse-ground groats |
| (3) | Milk (including flavoured milk, hot chocolate, latte) |
| (4) | Fermented milk drinks, e.g. yoghurts, kefir (natural or flavoured) |
| (5) | Fresh cheese curd products, e.g. cottage cheese, cream cheese, cheese-based puddings |
| (6) | White meat, e.g. chicken, turkey, rabbit |
| (7) | Fish |
| (8) | Legumes-based foods, e.g. beans, peas, soybeans, lentils |
| (9) | Fruit |
| (10) | Vegetables |
| **Components of non-Healthy-Diet-Index (nHDI)** | |
| (1) | White bread and bakery products, e.g. wheat bread, rye bread, wheat-rye bread, toast bread, bread rolls |
| (2) | White rice, white pasta, fine-ground groats, e.g. semolina, couscous |
| (3) | Fast foods, e.g. potato chips/French fries, hamburgers, pizza, hot-dogs |
| (4) | Fried foods, e.g. meat or flour-based foods such as dumplings, pancakes etc. |
| (5) | Butter as a bread spread or as an addition to your meals/ for frying/ for baking etc. |
| (6) | Lard as a bread spread, or as an addition to you meals/ for frying/ for baking etc. |
| (7) | Cheese (including processed cheese, blue cheese) |
| (8) | Cured meat, smoked sausages, hot-dogs |
| (9) | Red meat, e.g. pork, beef, veal, lamb, game |
| (10) | Sweets, e.g. confectionary, biscuits, cakes, chocolate bars, cereal bars, other |
| (11) | Tinned (jar) meats |
| (12) | Sweetened carbonated or still drinks |
| (13) | Energy drinks |
| (14) | Alcoholic beverages |

**Additional file 1:** **Table S3.** Missing data.

| Variable with missing value | N (100%) | Missing | |
| --- | --- | --- | --- |
|  |  | Count | Percent |
| SBP | 797 | 33 | 4.14 |
| DBP |  | 33 | 4.14 |
| BMI |  | 1 | 0.13 |
| WHR |  | 15 | 1.88 |
| Current smoking |  | 7 | 0.88 |
| Financial situation |  | 1 | 0.13 |
| Nutrition knowledge score |  | 13 | 1.63 |
| pHDI |  | 25 | 3.13 |
| nHDI |  | 16 | 2.00 |
